# Evodiamine as the Active Compound of Evodiae fructus to Inhibit Proliferation and Migration of Prostate Cancer through PI3K/AKT/NF-*κ*B Signaling Pathway

**DOI:** 10.1155/2022/4399334

**Published:** 2022-07-18

**Authors:** Yuhe Lei, Meiching Chan, Haiyan Liu, Wenyu Lyu, Lei Chen, Yinqin Zhong, Hua Gan, Mei Wang, Ming Qi, Yu Guo, Junshan Liu, Enxin Zhang

**Affiliations:** ^1^Shenzhen Hospital of Guangzhou University of Chinese Medicine, Shenzhen, 518034 Guangdong, China; ^2^Formula-Pattern Research Center, School of Traditional Chinese Medicine, Jinan University, Guangzhou, 510632 Guangdong, China; ^3^Second Clinical Medical College of Guangzhou University of Traditional Chinese Medicine, Guangzhou, 510006 Guangdong, China; ^4^College of Pharmacy, Jinan University, Guangzhou, 510632 Guangdong, China; ^5^School of Traditional Chinese Medicine, Jinan University, Guangzhou, 510632 Guangdong, China; ^6^School of Traditional Chinese Medicine, Southern Medical University, Guangzhou, 510510 Guangdong, China; ^7^Department of Pharmacy, Zhujiang Hospital, Southern Medical University, Guangzhou, 510280 Guangdong, China; ^8^Guangdong Provincial Key Laboratory of Chinese Medicine Pharmaceutics, Southern Medical University, Guangzhou 510510, China

## Abstract

Evodiae fructus (EF) is a traditional Chinese medicine which is widely used for the treatment of obesity, inflammation, cardiovascular disease, and diseases of the central nervous system. Recent studies have demonstrated the anticancer property of EF, but the active compounds of EF against prostate cancer and its underlying mechanism remain unknown. In this study, a network pharmacology-based approach was used to explore the multiple ingredients and targets of EF. Through protein-protein interaction (PPI), Gene Ontology (GO) enrichment, and Kyoto Encyclopedia of Genes and Genomes (KEGG) pathway enrichment analyses, the potential targets and corresponding ingredients of EF against prostate cancer cells were obtained. CCK8 and colony formation assays were performed to evaluate the antiproliferative effect of the active compounds on DU145 cells. Cell cycle analysis, Annexin V-FITC/PI staining assay, and Hoechst 33258 staining assay were used to explore the way of evodiamine-induced cell death. The capacities of cell migration after evodiamine treatment were evaluated by wound-healing assay. PharmMapper database was used to predict the potential targets of evodiamine against cancer cell migration. Western blot assay was performed to investigate the signaling pathway through which evodiamine inhibits cell proliferation and migration. The binding of evodiamine to PI3K and AKT was verified by molecular docking. As a consequence, 24 active compounds and 141 corresponding targets were obtained through a network pharmacology-based approach. The results of PPI analysis, GO enrichment, and KEGG pathway enrichment indicated that molecules in the PI3K/AKT/NF-*κ*B signaling pathway were the potential targets of EF against prostate cancer, and evodiamine was the potential active compound. *In vitro* study demonstrated that evodiamine displays antiproliferative effect on DU145 cells obviously. Evodiamine induces G_2_/M cell cycle arrest by Cdc25c/CDK1/cyclin B1 signaling. Additionally, evodiamine also promotes mitochondrial apoptosis and inhibits cell migration through PI3K/AKT/NF-*κ*B signaling in DU145 cells. In conclusion, evodiamine is the active compound of EF to inhibit proliferation and migration of prostate cancer through PI3K/AKT/NF-*κ*B signaling pathway, indicating that evodiamine may serve as a potential lead drug for prostate cancer treatment.

## 1. Introduction

Prostate cancer remains a huge challenge to men's health worldwide. It is reported that the incidence and mortality of prostate cancer ranks the second in males [1]. The treatments of prostate cancer include radiotherapy, chemotherapy, surgery, and hormonal therapy [2]. However, these treatments, particularly the standard androgen deprivation therapy (ADT), are not curative and easily result in resistance to therapeutic interventions [3]. Therefore, seeking efficient drugs with low toxicity is an urgent task for prostate cancer treatment.

Herbal medicine plays a major role in the prevention and treatment of cancers and other diseases worldwide, especially in Asian countries [4]. Numerous studies have demonstrated that a wide spectrum of traditional Chinese medicines (TCMs) possess anticancer properties, such as Scutellariae Barbatae Herba, Andrographis Herba, and Panax Ginseng C. A. Mey [5]. Screening natural compounds from TCM has attracted extensive attention. More and more promising compounds with potential anticancer activity, such as podophyllotoxin, camptothecin, and berberine, have been isolated from TCM [6, 7]. In recent years, “integrated pharmacology” (IP) has come into sight. It uses a network pharmacology approach to explore the synergistic effects of multiple ingredients, targets, and mechanisms of diseases based on multiple databases, which is perfect for TCM research [8]. Evodiae fructus (EF), a fruit of Tetradium ruticarpum, has been used in traditional Chinese herbal formulas for a long time. Numerous studies have revealed the therapeutic potential of EF on various diseases including obesity, inflammation, cardiovascular disease, cancers, and diseases of the central nervous system [9]. However, the active compounds of EF against prostate cancer and its underlying mechanism remain unknown.

Inhibiting cancer cell growth and metastasis constitute the major aspects in anticancer strategies. Cell migration is essential for tumor metastasis to colonize remote sites, frequently resulting in cancer deaths [10]. The phosphoinositide-3-kinase/protein kinase B (PI3K/AKT) signaling pathway participates in various biological processes including cell growth, survival, metabolism, invasion, and migration [11, 12]. PI3K/AKT signaling is aberrantly activated in a high proportion of prostate cancer patients [13]. PI3Ks are a class of heterodimers consisting of a catalytic subunit and a regulatory subunit [14]. AKT, a serine/threonine kinase, modulates the function of multiple substrates such as mTOR, NF-*κ*B, MDM2, and Bad [15]. Nuclear factor kappaB (NF-*κ*B), a transcription factor, translocates to the nucleus to facilitate oncogene transcription after activation in response to various stimuli [16]. Augmented phosphorylation of PI3K/AKT/NF-*κ*B signaling pathway has been confirmed to correlate to prostate cancer progression [17].

In this study, we identified evodiamine as the active compound of EF through a network pharmacology approach and evaluated the antiproliferative effects of evodiamine on prostate cancer DU145 cells. Further mechanistic study demonstrated that evodiamine induces mitochondrial apoptosis and inhibits migration of prostate cancer cells through PI3K/AKT/NF-*κ*B signaling pathway. This study will provide a rationale for using evodiamine as the potential lead drug for prostate cancer treatment.

## 2. Materials and Methods

### 2.1. Reagents and Antibodies

The chemical compounds evodiamine, rutaecarpine, berberine, quercetin, and *β*-sitosterol were purchased from Baoji Herbest Bio-Tech Co., Ltd. (Baoji, Shanxi, China). The Cell Counting Kit-8 (CCK8) was obtained from Good Laboratory Practice Bioscience (California, Montclair, USA). The Hoechst 33258 was supplied by Beijing Solarbio Science & Technology Co., Ltd. (Beijing, China). The Annexin V-FITC/PI staining assay kit was purchased from Dalian Meilun Biotechnology Co., Ltd. (Dalian, Liaoning, China). Propidium iodide, crystal violet, and RNase were obtained from Sigma-Aldrich (St. Louis, MO, USA). The BCA protein quantitation assay kit was supplied by KeyGEN BioTECH (Nanjing, Jiangsu, China). The primary antibodies against CDK1, p-CDK1^Thr14^, cyclin B1, p-Cdc25C^Ser216^, Bax, Bcl-2, AKT, *β*-actin, p-AKT^Ser473^, NF-*κ*B, PARP, and PI3K were purchased from Proteintech (Wuhan, Hubei, China). The anti-PI3K p85 (phospho Y458)+PI3 kinase p55 (phospho Y199) antibody [PI3KY458-1A11] was supplied by Abcam (Shanghai, China). The primary antibodies against pro- and cleaved-caspase 3/9 were obtained from Abscitech (Shanghai, China). The primary antibodies against p-NF-*κ*B^Ser536^ and the secondary antibodies anti-rabbit IgG and antimouse IgG were obtained from Cell Signaling Technology (Danvers, MA, USA). Other reagents were obtained from Sigma-Aldrich (St. Louis, MO, USA).

### 2.2. Compounds and Targets Screening of EF

The traditional Chinese medicine system pharmacology (TCMSP) database, a unique system pharmacology platform of Chinese herbal medicines [18], was used to search for the ingredients and targets of EF. To obtain the active compounds of all the ingredients according to ADME (absorption, distribution, metabolism, and excretion) properties, we selected the compounds which meet the requirements of both oral bioavailability ≥ 30% [15] and drug − likeness (DL) ≥ 0.18. The information of the active compounds and their related targets was collected for further research. UniProt (https://www.uniprot.org) was used to convert protein names to gene symbols.

### 2.3. Prediction of Potential Targets of EF on Prostate Cancer

The known therapeutic targets of prostate cancer were obtained from the GeneCards database (https://www.genecards.org). Key terms “prostate cancer,” “prostate adenocarcinoma,” and “prostatic cancer” were retrieved, and the requirement of relevance score ≥ 5 was set. After getting the disease-related genes, we selected the target genes at the intersection of EF and prostate cancer (E&P), which were regarded as potential target genes of EF against prostate cancer. The chemical compounds corresponding to E&P targets were considered as therapeutic components of EF against prostate cancer.

### 2.4. Drug-Compound-Target-Disease Network Construction

Based on the active compounds and corresponding targets of E&P, we employed cytoscape (v3.8.0, Agilent Technologies Company, USA) to visualize the drug-compound-target-disease network. Each node in the network represents a drug, disease, target, or compound. Each line in the network represents the connection of drug-compound, compound-target, and target-disease.

### 2.5. Protein-Protein Interaction (PPI) Network Analysis

The PPI network of E&P targets was obtained from the STRING database (https://string-db.org/). Gene symbols of the targets were submitted to the STRING database, and the required interaction score is high confidence (≥0.700). The bitmap image and simple tabular text output were downloaded from this website. After enrichment of all the nodes, the top 20 targets, which were considered to be of significance in the PPI network, were selected.

### 2.6. Gene Ontology (GO) Enrichment and KEGG Pathway Enrichment

GO enrichment and KEGG pathway enrichment analyses were based on the Bioconductor software (http://bioconductor.org/). We used the R statistical programming language (cluster-profiler version 4.1) to load the Bioconductor source and gene symbols of E&P. The results of GO enrichment and KEGG enrichment were considered significant when *P* value < 0.05. The top 20 targets in GO and KEGG enrichment were displayed in barplot and dotplot.

### 2.7. Cell Line and Cell Culture

The human prostate cancer cell line DU145 was obtained from the Chinese Academy of Sciences Cell Bank (Shanghai, China). Cells were cultured in RPMI 1640 supplemented with 10% fetal bovine serum (FBS) and 1% penicillin-streptomycin (PS) in 5% CO_2_ containing incubator at 37°C. The DU145 cell line was identified by short tandem repeat (STR) profiling and tested for mycoplasma (Genetic Testing Biotechnology Inc., Suzhou, Jiangsu, China).

### 2.8. Cell Viability Assay

Viability of DU145 cells was measured using the Cell Counting Kit-8 (CCK8) assay. Cells (5,000/well) were seeded into 96-well plates and cultured overnight. After treatment with different concentrations of evodiamine, rutaecarpine, berberine, quercetin, and *β*-sitosterol for 72 h, the cells were exposed to 100 *μ*L/well-diluted CCK8 solution. Then, the light absorbance was detected at 450 nm by a microplate reader (Beckman Coulter Inc., USA).

### 2.9. Colony Formation Assay

DU145 cells were cultured in 6-well plates (2000 cells/well) for 24 h. Then, the cells were exposed to evodiamine at the concentrations of 0, 1.25, 2.5, and 5.0 *μ*M for 48 h. After washing with phosphate-buffered saline (PBS), cells were cultured in the fresh medium which was replaced every three days. After ten days, the cells were fixed in 75% alcohol for 10 min at 4°C and stained with 1% crystal violet for 30 min. After washing twice with PBS, the number of colonies > 0.5 mm in diameter was counted manually, and the images of colonies were photographed.

### 2.10. Cell Cycle Analysis

DU145 cells (2 × 10^5^/well) were seeded in 6-well plates and cultured overnight. After treatment with evodiamine at the concentrations of 0, 1.25, 2.5, and 5.0 *μ*M for 24 h, cells were fixed and permeabilized with precooled 75% ethanol at 4°C overnight. Then, cells were incubated with PI (0.2 mg/mL) and RNase (0.1 mg/ml) for 15 min in the dark at room temperature. The Epics XL Flow cytometry (Beckman Coulter, USA) was used to detect the PI fluorescence. The phase distribution of cell cycle was analyzed by the ModFit LT v3.1 software (Verity Software House, Inc.)

### 2.11. Annexin V-FITC/PI Staining Assay

Cell apoptotic rate was measured by Annexin V-FITC/PI staining assay. Cells (1 × 10^5^/well) were seeded in 6-well plates and cultured overnight. After treatment with evodiamine at the concentrations of 0, 1.25, 2.5, and 5.0 *μ*M for 24 h, DU145 cells were collected and stained with Annexin V-FITC for 15 min and PI for 5 min in darkness at room temperature. Then, Epics XL flow cytometer (Beckman Coulter Inc.) was used to measure the cell apoptotic rates (excitation = 488 nm and emission = 525 nm for Annexin V-FITC; excitation = 488 nm and emission = 620 nm for PI). The data was quantified using the FlowJo v7.6 software (FlowJo LLC).

### 2.12. Hoechst 33258 Staining Assay

DU145 cells (2 × 10^5^/well) were seeded into 6-well plates. After culture for 24 h, cells were treated with evodiamine at the concentrations of 0, 1.25, 2.5, and 5.0 *μ*M for 24 h. Then, PBS was added to wash the cells. After fix for 30 min, the cells were stained with Hoechst 33258 (1 mg/mL) for 30 min at 37°C. A fluorescence microscope (Carl Zeiss, Jena, Germany) was applied to observe the nuclear morphology of DU145 cells.

### 2.13. Western Blot Analysis

Following treatment with different concentrations of evodiamine for 24 h, DU145 cells were collected using trypsin. Then, the RIPA lysis buffer (containing 1 mM PMSF, 1× phosphatase inhibitor, and 1× protease inhibitor) was added to obtain the total cellular protein. The BCA assay was performed to quantify the protein concentration. Proteins (30 *μ*g/lane) were separated by 12% SDS-PAGE gels and then transferred to PVDF membranes. The membranes were blocked with 5% skimmed milk at room temperature for 1 h. After incubation with primary antibody overnight at 4°C and secondary antibody for 1 h at room temperature, the protein bands were visualized by ECL detection kit (Millipore, Merck KGaA) and quantified using the ImageJ software v1.8.0 (National Institutes of Health). *β*-Actin was used as the loading control.

### 2.14. Wound-Healing Assay

DU145 cells (2 × 10^5^/well) were seeded into 6-well plates and cultured. After reaching 80% confluency, cells were scratched in a straight line with 200 *μ*L pipette tips. Then, different concentrations of evodiamine (0.5 and 1.0 *μ*M) in serum-free medium were added. Images of cells treated with different time (0, 6, 12, and 24 h) were acquired with an Olympus IX70 inverted microscope (Shinjuku, Tokyo, Japan).

### 2.15. Prediction of Potential Targets of Evodiamine on Cell Migration

The potential targets of evodiamine were obtained from PharmMapper database (http://www.lilab-ecust.cn/pharmmapper/), an updated integrated pharmacophore matching platform that can be used to identify potential target candidates for given small molecules using a pharmacophore mapping approach [19]. The chemical structure of evodiamine submitted to the PharmMapper website was downloaded from the PubChem database (https://pubchem.ncbi.nlm.nih.gov). The known therapeutic targets of cancer metastasis were obtained from the GeneCards database. Key term “cancer cell migration” was retrieved, and the requirement of relevance score ≥ 20 was set. After getting the cell migration-related genes, we selected the targets at the intersection of evodiamine and cell migration (E&M), which were regarded as potential target genes of evodiamine against cell migration.

### 2.16. Molecular Docking

PDB database (https://www.pdbus.org/) was used to search for the conformational information of PIK3CG (PDB ID: 6AUD) and AKT1 (PDB ID: 4GV1). After removing irrelevant small molecules in the crystal structure by the Pymol 2.1 software and adding with Kollman atom charges, solvation parameters, and polar hydrogens by AutoDock Tools (1.5.6 software), PIK3CG and AKT1 were used as the receptors. The PubChem database (https://pubchem.ncbi.nlm.nih.gov) was used to obtain the chemical structure of evodiamine. Then, we minimized the energy of evodiamine by Chem3D and converted it into mol2 format. After adding with atomic charge and assigning an atomic type by AutoDock Tools, evodiamine was used as the ligand. Then, the ligand and receptors were imported into AutoDock 4.2 to start the docking process. The free energy of binding in the receptor was calculated through Lamarckian genetic algorithm. Then, Pymol 2.1 was used to visualize the binding of evodiamine to PIK3CG and AKT1.

### 2.17. Statistical Analysis

All experiments were performed in triplicate. Results are presented as the mean ± standard deviation (SD). For the statistical analysis, GraphPad Prism 7.0 (GraphPad Software Inc.) was used to evaluate one-way analysis of variance (ANOVA) followed by Tukey's post hoc test. *P* < 0.05 was considered statistically significant.

## 3. Results

### 3.1. Screening of Active Compounds and Potential Targets of EF against Prostate Cancer

**Figure 1:**
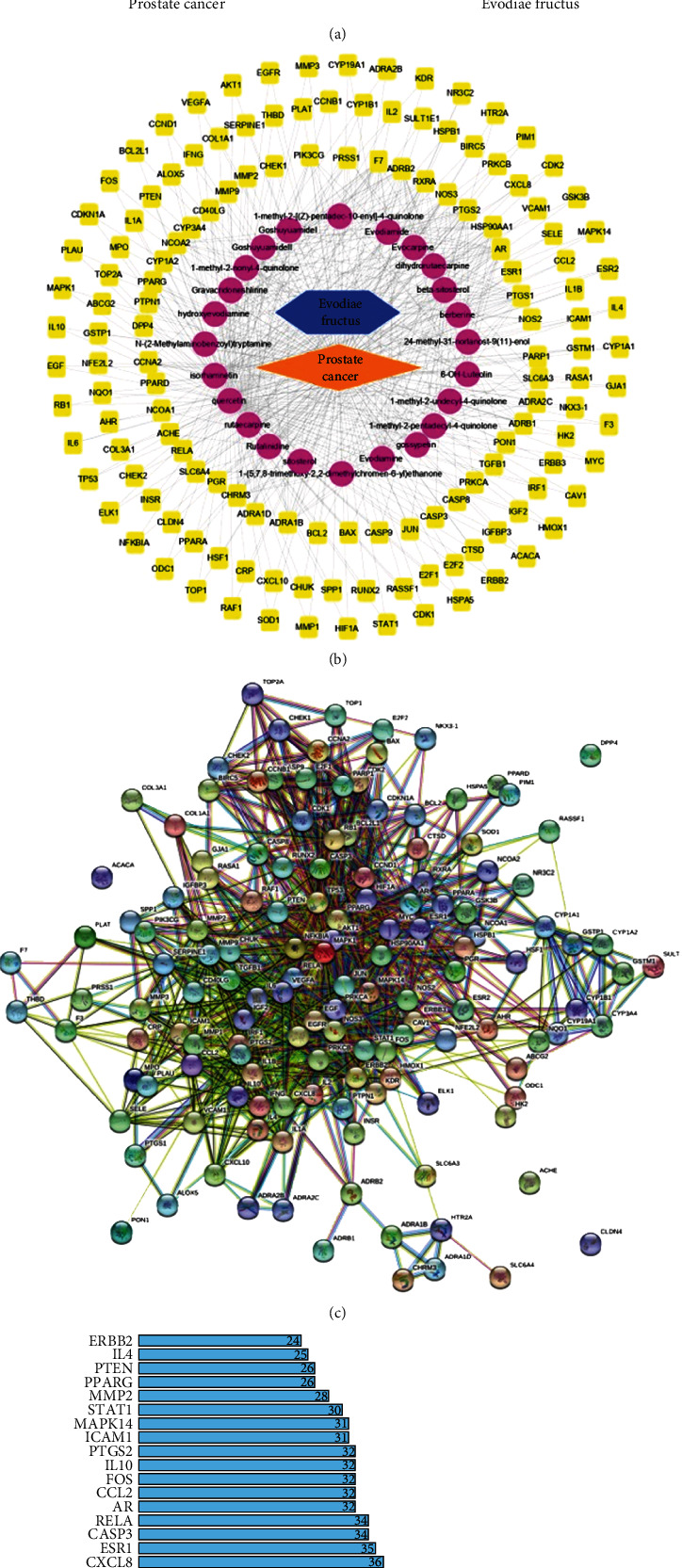
The construction of drug-compound-target-disease network and PPI network. (a) Venn diagram displays the overlap between the prostate cancer-related targets and the potential targets of EF. (b) The construction of drug-compound-target-disease network. The orange rhombus represents diseases. The blue hexagon represents drugs. The purple ovals represent active compounds. The yellow rectangles represent target genes. (c) PPI network of 141 target genes of E&P. Each node represents the E&P targets. Each line represents the interaction between two targets. (d) The top 30 enriched targets in the PPI network were displayed in a barplot.

After retrieval in TCMSP, 176 compounds of EF and 1504 related targets were obtained. We selected 30 active compounds that met the requirements of both oral bioavailability ≥ 30% [15] and drug − likeness (DL) ≥ 0.18, as well as 197 corresponding targets. They form 501 compound-target connections. After retrieval in the GeneCards database, 11719 prostate cancer-related targets were collected and 2340 of them met the requirement of relevance score ≥ 5. We obtained the intersections between 197 drug targets and 2340 disease targets, resulting in 141 E&P targets corresponding to 24 compounds (Figure 1(a)). These 141 genes were regarded as potential targets through which EF exerts its antiprostate cancer effects, and 24 compounds were regarded as candidate components. Then, the cytoscape software was used to establish drug-compound-target-disease network. As shown in Figure 1(b), 24 drug-compound, 346 compound-target, and 141 target-disease connections were created in a network, which integrally illustrated the anticancer activity of EF characterized by multi-ingredients, multitargets, and synergistic effects. The PPI analysis of E&P targets was performed by STRING. Totally, 141 target genes of E&P were searched in the STRING database, and a total of 1126 PPI connections were generated (Figure 1(c)). According to the frequency of each node and the combined score between two nodes, the top 30 enriched targets were displayed in a barplot (Figure 1(d)). The results demonstrated that AKT1, TP53, MAPK1, and other targets are associated with the antiprostate cancer effects of EF.

### 3.2. Prediction of Antiprostate Cancer Mechanism by GO and KEGG Enrichment

**Figure 2:**
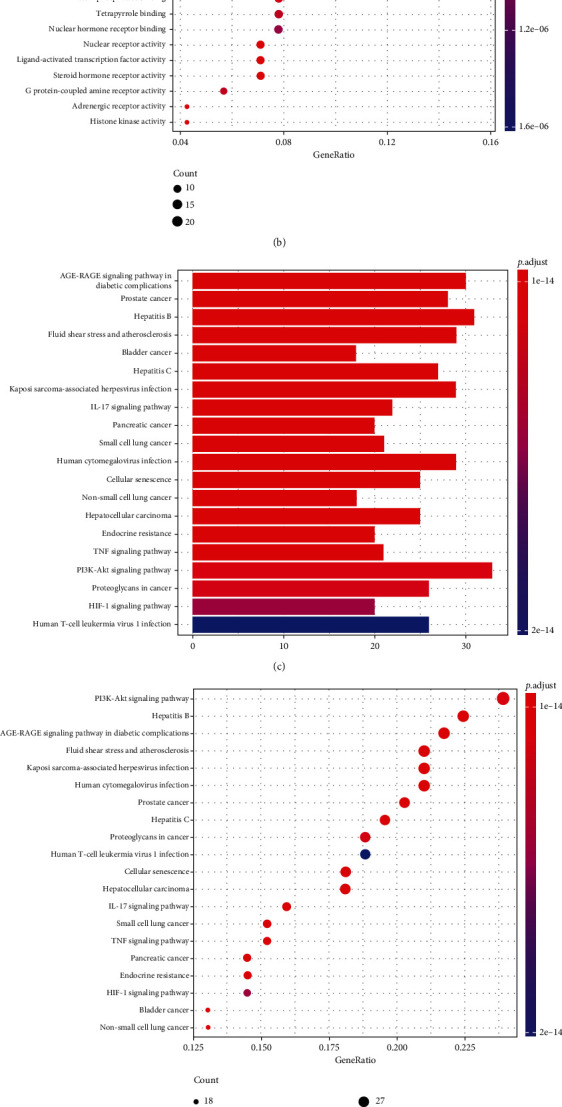
The GO functional enrichment and KEGG pathway enrichment. (a) The barplot of top 20 items identified by GO functional enrichment. (b) The dotplot of top 20 items identified by GO functional enrichment. (c) The barplot of top 20 items identified by KEGG pathway enrichment. (d) The dotplot of top 20 items identified by KEGG pathway enrichment. The color of the bubble and column is associated with the *P* value, and the size of bubble is related to the ratio of target gene. (e) PI3K/AKT signaling pathway enriched in KEGG pathway analysis.

The results of GO functional enrichment were displayed in a barplot (Figure 2(a)) and a dotplot (Figure 2(b)). According to the results, the main molecular functions of the targets include DNA-binding transcription factor, nuclear receptor, and ligand-activated transcription factor. The results of KEGG pathway enrichment in a barplot (Figure 2(c)) and a dotplot (Figure 2(d)) showed that PI3K/AKT and AGE-RAGE signaling pathways were the potential pathways mediating the antiprostate cancer effects of EF. The “prostate cancer” listed in the second in Figure 2(c) and the seventh in Figure 2(d) also confirmed the cancer type which EF is more likely to influence on. Since AKT is the most significant target protein in the PPI network (Figure 1(d)), we focused on the PI3K/AKT signaling pathway, which is closely related to prostate cancer initiation and progression [20]. From the results of PI3K/AKT signaling pathway enriched in KEGG (Figure 2(e)), we predicted that PI3K/AKT/NF-*κ*B signaling pathway may participate in the inhibitory effects of EF on prostate cancer since NF-*κ*B is a key transcription factor downstream of PI3K/AKT to mediate prostate carcinogenesis [21], which is also consistent with the results of GO enrichment featuring transcription factor binding (Figures 2(a) and 2(b)). Based on these results, we suggested that evodiamine, which targets PI3K, may be the active ingredient of EF for prostate cancer treatment.

### 3.3. Evodiamine Displays Obvious Antiproliferative Effect on DU145 Cells

**Figure 3:**
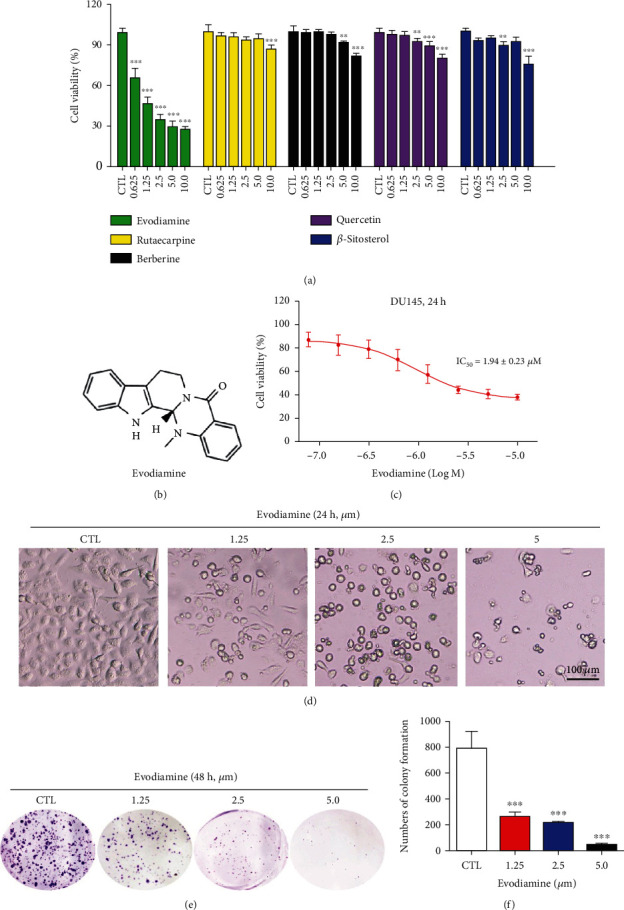
Evodiamine displays antiproliferative effect on DU145 cells. (a) DU145 cells were treated with 5 potential active compounds at different concentrations (0, 0.625, 1.25, 2.5, 5.0, and 10.0 *μ*M) for 72 h. The cell viability was detected by CCK8 assay. Among these compounds, evodiamine (chemical structure in Figure 3(b)) displays the most potent cytotoxicity. Data are presented as mean ± SD (*n* = 3). ^∗∗^*P* < 0.01 and ^∗∗∗^*P* < 0.001 versus the control group. (c) DU145 cells were treated with different concentrations of evodiamine for 24 h. The cell viability was detected by CCK8 assay. The curve indicated that E2 exerts antiproliferative effect on DU145 cells in a dose-dependent manner. (d) Representative images of CCK8 assay. Original magnification: 100x; scale bar: 100 *μ*m. (e) DU145 cells were exposed to evodiamine at the concentrations of 0, 1.25, 2.5, and 5.0 *μ*M for 48 h and then cultured in fresh medium which was replaced every three days. After ten days, the colonies of DU145 cells were visualized by crystal violet staining. (f) The numbers of colonies of DU145 cells were counted and presented as mean ± SD (*n* = 3). ^∗∗∗^*P* < 0.001 versus the control group.

Among the 24 potential active compounds of EF against prostate cancer, 5 of them were selected to evaluate their cytotoxicity on DU145 cells. As shown in Figure 3(a), evodiamine (chemical structure in Figure 3(b)) displays more potent antiproliferative effect than rutaecarpine, berberine, quercetin, and *β*-sitosterol on DU145 cells. Evodiamine, an indoloquinazoline alkaloid isolated from EF, was reported to display cytotoxicity on various types of cancers [22, 23]. Subsequently, the antiproliferative effect of evodiamine on DU145 cells was further examined. As shown in Figure 3(c), the viability of DU145 cells was significantly inhibited in a dose-dependent manner after treatment with evodiamine for 24 h, with the IC_50_ value of 1.94 ± 0.23 *μ*M. The evodiamine-induced cell morphology changes under the microscope were presented in Figure 3(d), which indicated that evodiamine has potent cytotoxicity on DU145 cells. In addition, the long-term efficacy of evodiamine on DU145 cell survival was evaluated by colony formation assay. The results demonstrated that evodiamine inhibits the cell proliferation in a dose-dependent way (Figures 3(e) and 3(f)).

### 3.4. Evodiamine Induces G_2_/M Cell Cycle Arrest in DU145 Cells

**Figure 4:**
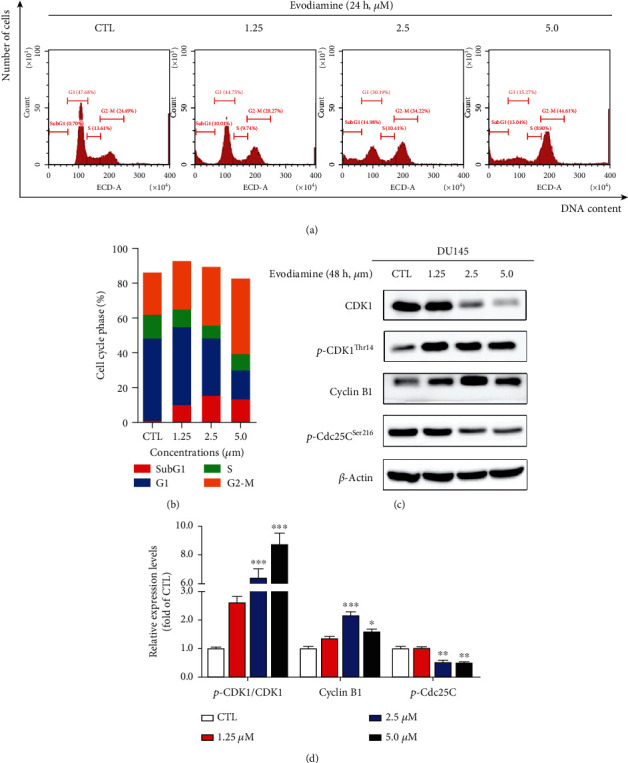
Evodiamine induces G_2_/M cell cycle arrest in DU145 cells. (a) After treatment with evodiamine (0, 1.25, 2.5, and 5.0 *μ*M) for 24 h, the cell cycle distributions were analyzed by flow cytometry. The cell population in the G_2_/M phase significantly augmented in a dose-dependent manner. (b) The cell populations were quantified using Prism. Each column represents the cell population in different phases (*n* = 3). (c) DU145 cells were treated with evodiamine (0, 1.25, 2.5, and 5.0 *μ*M) for 24 h. The protein expression levels of CDK1, p-CDK1^Thr14^, cyclin B1, and p-Cdc25C^Ser216^ were detected by Western blot. *β*-Actin was used as the loading control. Evodiamine-induced G_2_/M cell cycle arrest is associated with upregulation of p-CDK1^Thr14^/CDK1 and cyclin B1 and downregulation of p-Cdc25C^Ser216^. (d) Quantitative analysis of the relative protein expression. Data are presented as the mean ± SD (*n* = 3). ^∗^*P* < 0.05, ^∗∗^*P* < 0.01, and ^∗∗∗^*P* < 0.001 versus the control group.

To determine whether the inhibitory effect of evodiamine on DU145 cells is related to cell cycle arrest, flow cytometry was used to analyze the content of DNA in various stages of DU145 cells. As shown in Figures 4(a) and 4(b), compared with the control group, the cell population in G_2_/M phase significantly increased from 24.48% to 44.61% following 5 *μ*M evodiamine treatment, suggesting that evodiamine induces G_2_/M cell cycle arrest in DU145 cells. CDK1 and cyclin B1 are key regulators involved in the G_2_/M transition by forming the CDK1/cyclin B1 complex [24]. Cdc25C activates the CDK1 complex through CDK1^Tyr15^ and CDK1^Thr14^ dephosphorylation [25]. As shown in Figures 4(c) and 4(d), evodiamine decreased the level of p-Cdc25C^Ser216^ in a dose-dependent manner, indicating that the activation of Cdc25c is inhibited by evodiamine. As a consequence, the inactive Cdc25C can no longer dephosphorylate p-CDK1^Thr14^, which makes p-CDK1^Thr14^ and cyclin B1 accumulation to block the G_2_/M transition. These results indicated that evodiamine induces G_2_/M cell cycle arrest in DU145 cells through regulating Cdc25c/CDK1/cyclin B1 signaling pathway.

### 3.5. Evodiamine Induces Mitochondrial Apoptosis in DU145 Cells

**Figure 5:**
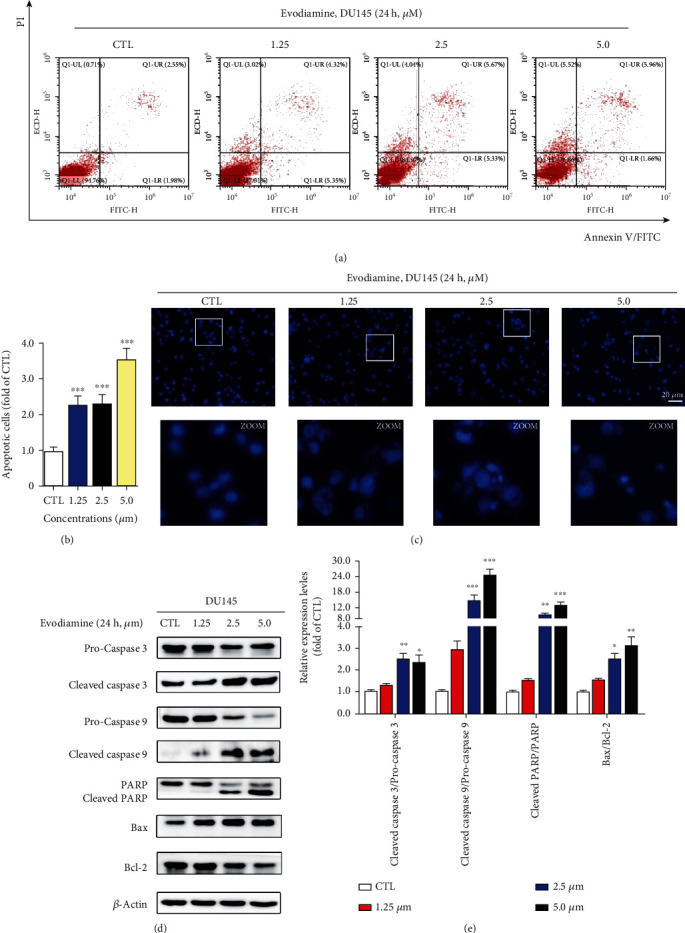
Evodiamine induces mitochondrial apoptosis in DU145 cells. (a) After evodiamine treatment (0, 1.25, 2.5, and 5.0 *μ*M) for 24 h, the apoptotic rate of DU145 cells was measured by PI/Annexin V-FITC staining assay. Representative images are shown. Evodiamine induces cell apoptosis in a dose-dependent manner. (b) Quantitative data of evodiamine-induced apoptotic cells. Data are presented as the mean ± SD (*n* = 3). ^∗∗∗^*P* < 0.001 versus the control group. (c) Apoptotic morphological changes observed by Hoechst 33258 staining assay after evodiamine treatment (0, 1.25, 2.5, and 5.0 *μ*M) for 24 h. Original magnification: 200x; scale bar: 20 *μ*m. (d) After evodiamine treatment (0, 1.25, 2.5, and 5.0 *μ*M) for 24 h, the expression levels of apoptosis-related proteins including pro- and cleaved-caspases 3/9, PARP, cleaved-PARP, Bax, and Bcl-2 were detected by Western blotting. *β*-Actin was used as the loading control. (e) The quantitative data of relative protein expression shown as the mean ± SD (*n* = 3). ^∗^*P* < 0.05, ^∗∗^*P* < 0.01, and ^∗∗∗^*P* < 0.001 versus the control group.

To investigate whether evodiamine-induced cell death is attributed to cell apoptosis, the Annexin V-FITC/PI staining assay was performed using flow cytometry. Figures 5(a) and 5(b) demonstrated that evodiamine treatments at different concentrations (1.25, 2.5, and 5.0 *μ*M) increased the number of apoptotic cells. The apoptotic cell ratio (early apoptotic stage plus late stage) in the 5.0 *μ*M treatment group is almost 4 times of that of the control group. In addition, the Hoechst 33258 staining assay was performed to observe nuclear morphological changes in DU145 cells. As shown in Figure 5(c), following different concentrations of evodiamine treatment for 24 h, cells emit bright blue fluorescence representing nuclear condensation and DNA fragmentation which are the typical characteristics of cell apoptosis. The fluorescence intensity in evodiamine-treated cells is significantly higher than that of the control group. These results provided evidence for the induction of apoptosis by evodiamine. A Western blotting assay was applied to detect the expression levels of apoptosis-related proteins. Figures 5(d) and 5(e) displayed that evodiamine upregulates the ratio of cleaved-caspase 3/procaspase 3, cleaved-caspase 9/procaspase 9, and cleaved PARP/PARP. Moreover, evodiamine significantly changed the ratio of Bax/Bcl-2. It is well recognized that the interaction between apoptotic promotor Bax and apoptotic inhibitor Bcl-2 determines the fate of cell towards mitochondrial apoptosis [26]. These results demonstrated that evodiamine induces mitochondrial apoptosis in DU145 cells.

### 3.6. Evodiamine May Inhibit DU145 Cell Migration through PI3K Signaling Pathway

**Figure 6:**
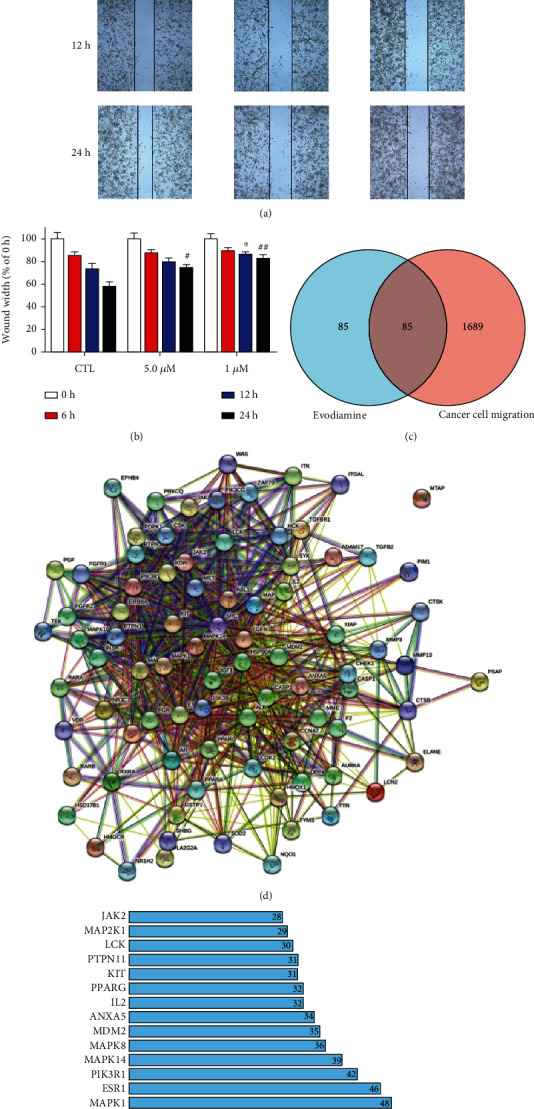
Evodiamine may inhibit DU145 cell migration through PI3K signaling pathway. (a) The migratory properties of DU145 cells were analyzed by wound-healing assays. Original magnification: 100x. (b) The relative wound width was analyzed using GraphPad Prism 7.0. Data are presented as the mean ± SD (*n* = 3). ^∗^*P* < 0.05, ^#^*P* < 0.05, and ^##^*P* < 0.01 versus the control group. (c) Venn diagram displays the overlap between the migration-related targets and the potential targets of evodiamine. (d) PPI network of 85 target genes of E&M. Each node represents the E&M targets. Each line represents the interaction between two targets. (e) The top 20 enriched targets in the PPI network were displayed in a barplot.

To evaluate the capacity of cell migration, the wound-healing assay was applied. As shown in Figures 6(a) and 6(b), evodiamine treatments at different concentrations (0.5 and 1.0 *μ*M) inhibited wound closure in a time-dependent manner. To further predict the targets of evodiamine against cell migration, we used PharmMapper database to obtain targets of evodiamine and GeneCards database to obtain cell migration-related targets. As a result, 170 drug targets and 1774 cancer cell migration targets which met the requirement of relevance score ≥ 20 were collected. The 85 targets (E&M) at the intersection between drug targets and migration targets were regarded as potential antimigration targets of evodiamine (Figure 6(c)). The PPI analysis of E&M targets was performed by STRING. Totally, 85 gene symbols of E&M were searched in the STRING database, and a total of 844 PPI connections were generated (Figure 6(d)). According to the frequency of each node and the combined score between two nodes, the top 20 enriched targets were displayed in a barplot (Figure 6(e)), which represent the most probable antimigration targets of evodiamine. Since PI3K turned out to be both antiprostate cancer and antimigration targets of evodiamine, we predicted that evodiamine may inhibit DU145 cell migration through PI3K signaling pathway.

### 3.7. Evodiamine Exerts Antiprostate Cancer Effects through PI3K/AKT/NF-*κ*B Signaling Pathway

**Figure 7:**
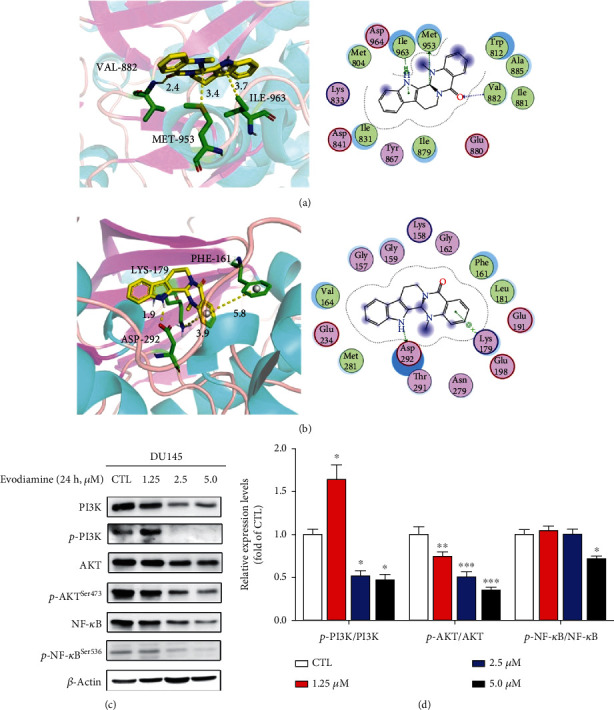
Evodiamine exerts antiprostate cancer effects through PI3K/AKT/NF-*κ*B signaling pathway. (a) The binding mode of evodiamine with PI3K (left: 3D structure; right: 2D structure). The yellow structure is evodiamine, and the green structure represents the binding site of PI3K. (b) The binding mode of evodiamine with AKT (left: 3D structure; right: 2D structure). The yellow structure is evodiamine, and the green structure represents the binding site of AKT. (c) After evodiamine treatment (0, 1.25, 2.5, and 5.0 *μ*M) for 24 h, the expression levels of p-PI3K, PI3K, p-AKT^Ser473^, AKT, p-NF-*κ*B^Ser536^, and NF-*κ*B were detected by Western blotting. *β*-Actin was used as the loading control. (d) The quantitative data of relative protein expression shown as the mean ± SD (*n* = 3). ^∗^*P* < 0.05, ^∗∗^*P* < 0.01, and ^∗∗∗^*P* < 0.001 versus the control group.

Previous studies demonstrated that AKT plays a central role in mediating the antiprostate cancer effect of EF, and PI3K was predicted to be the potential target of evodiamine to inhibit proliferation and migration of prostate cancer. In addition, the activation of PI3K/AKT/NF-*κ*B signaling pathway was confirmed to be closely related to pathogenesis of prostate cancer [21]. Therefore, the molecular docking of evodiamine to PI3K and AKT was performed. As shown in Figure 7(a), the binding free energy of evodiamine to PI3K is -6.77 kcal/mol, indicating a good binding affinity. The interaction type includes hydrogen bonds, hydrophobic interactive, and *π*-stacking. Evodiamine binds to active amino acid residues of PI3K including ILE-963, MET-953, and VAL-882. Evodiamine belongs to a type of polycyclic compound with potent hydrophobic property, which interacts with hydrophobic residues of PI3K including ILE-963, ILE-879, MET-804, TRP-812, and ILE-831 through hydrophobic effect. Moreover, evodiamine binds to MET-953 and VAL-882 residues of PI3K through hydrogen bond interaction. The average hydrogen bond distance is 3.4 Å and 2.4 Å, which is lower than the conventional hydrogen bond distance 3.5 Å. As shown in Figure 7(b), the binding free energy of evodiamine to AKT is -6.82 kcal/mol, indicating a good binding affinity. Evodiamine binds to active amino acid residues of AKT including LYS-179, PHE-161, and ASP-292. Evodiamine is an indoloquinazoline alkaloid with six-membered rings that can form strong hydrophobic interactions with the pocket amino acids of AKT such as ILE-963, ILE-879, MET-804, TRP-812, and ILE-831. Additionally, evodiamine can bond to ASP-292 residues of AKT through hydrogen bond interaction. The average hydrogen bond distance is 2.92, which is much lower than the conventional hydrogen bond distance 3.5 Å. The results of molecular docking demonstrated that evodiamine is a potential active molecule targeting PI3K and AKT. The results of Western blot also confirmed the inhibition of PI3K/AKT/NF-*κ*B signaling pathway by evodiamine. As shown in Figures 7(c) and 7(d), the expression levels of p-PI3K/PI3K, p-AKT^Ser473^/AKT, and p-NF-*κ*B^Ser536^/NF-*κ*B were decreased following evodiamine treatment, indicating that inhibition of PI3K/AKT/NF-*κ*B signaling by evodiamine may result in proliferation and migration inhibition of DU145 cells.

## 4. Discussion

TCM, which is widely used in clinics especially in Asia and Africa, has displayed the great potential in the prevention and treatment of cancers and other diseases [4]. Isolation of active compounds from TCM is an important strategy for drug discovery [27]. The discovery of vinblastine and vincristine was the beginning of developing anticancer drugs from natural resources [28]. It has been reported that approximately 80% of small molecule anticancer drugs are natural products and their derivatives [29]. The multiple pharmacological properties of natural compounds provided a basis for the mechanistic study of their biological functions. TCMSP is a unique system pharmacology platform of Chinese herbal medicine that captures the relationships among drugs, targets, and diseases [18]. Through target prediction in TCMSP and PPI analysis in STRING database, AKT was identified as the most central target in antiprostate cancer effects of EF. Through GO and KEGG enrichment, PI3K/AKT was predicted as the most likely signaling pathway by which EF displays its antiprostate cancer effects. From 5 potential active compounds of EF, evodiamine was verified to possess the most potent cytotoxicity against DU145 cells. In addition, the results of target prediction showed that PI3K is the potential target of evodiamine against prostate cancer and cell migration. Hence, we suggested that evodiamine is the active compound of EF to inhibit proliferation and migration of prostate cancer through PI3K/AKT signaling pathway. To predict the downstream substrates of AKT, we focused on the transcription factor NF-*κ*B, which is closely related to tumorigenesis and tumor progression [30]. The results of molecular docking demonstrated a good binding affinity between evodiamine and PI3K, as well as evodiamine and AKT. The inhibition of PI3K/AKT/NF-*κ*B by evodiamine *in vitro* was also verified by Western blot. However, evodiamine was selected as the most active ingredient of EF against prostate cancer but not the only one. The active ingredients of herbal medicine are multiple and complex. TCM exerts effects on disease through multi-ingredients, multitargets, and synergetic way. Hence, the complex connections between EF and prostate cancer indicate that multiple possible mechanism participate in this process, which need further investigation. This research provides an example for the exploration of pharmacological mechanism of TCM. However, *in vivo* studies are needed to further confirm the inhibitory effects of evodiamine on tumor growth and tumor metastasis through PI3K/AKT/NF-*κ*B signaling pathway.

Natural compounds and their derivatives exert anticancer effects via multiple mechanisms. In traditional herbal medicine, EF has been used for the treatment of headaches, abdominal pain, and menorrhalgia [31]. Through activity screening and mechanistic study, evodiamine was identified as one of the major bioactive components of EF against various types of cancers including colon cancer [32], hepatocellular carcinoma (HCC) [33], lung cancer [34], and melanoma [35]. It is reported that different mechanisms are involved in the anticancer effects of evodiamine such as induction of apoptosis, cell cycle arrest, inhibition of invasion and metastasis [36]. The targets of evodiamine include topoisomerases, aryl hydrocarbon receptor (AhR), and transient receptor potential cation channel subfamily V member 1 (TRPV1) in the treatment of different types of cancers [37]. Various signaling pathways participate in the evodiamine-induced cancer cell apoptosis such as mTOR signaling [38], STAT3 signaling [39], and Bax/Bcl-2 [40]. Meanwhile, evodiamine repressed the EMT of gastric cancer stem cells by inhibiting Wnt pathway [41]. The inactivation of the PI3K/AKT signaling pathway induced by evodiamine was previously verified to result in cell apoptosis in pancreatic cancer [42, 43], glioma [44], and melanoma [45], which is consistent with our present study. This is the first time to report that inhibition of PI3K/AKT/NF-*κ*B signaling pathway is associated with the antiprostate cancer effect of evodiamine, making it a promising therapeutic lead drug for prostate cancer treatment. Currently, numerous efforts have been made to explore small molecular inhibitors targeting the PI3K/AKT signaling pathway to block cancer growth and metastasis. However, the clinical efficacies of these inhibitors are limited since the activation of the PI3K family occurs through complex mechanisms [46]. Hence, combination of PI3K/AKT inhibitors and other cancer treatments has been proposed to solve the therapeutic dilemma [47]. Future research may focus on the combination of evodiamine and other cancer therapies to improve the treatment efficacy.

However, the poor bioavailability and potential toxicity limit the clinical application of evodiamine. Evodiamine inhibits the activities of metabolic enzymes such as cytochrome P450, leading to cytotoxic effects [48]. Moreover, the safety concern exists as the precise target of evodiamine is unknown and the excessive inhibition of PI3K/AKT/NF-*κ*B pathway may bring about side effects. Hence, evodiamine treatment may be more potent but not necessarily more effective than EF treatment, which needs further evaluation. Currently, a number of novel drug delivery systems have been designed to improve the bioavailability and minimize side effects of low-solubility natural medicines [49]. Further research aiming to enhance the anticancer effects of evodiamine would prove beneficial.

## 5. Conclusion

In conclusion, this study demonstrated that evodiamine is the active compound of Evodiae fructus to inhibit proliferation and migration of prostate cancer through PI3K/AKT/NF-*κ*B signaling pathway. This study provides a rationale of using evodiamine as the potential lead drug for prostate cancer treatment.

## Data Availability

The data supporting the conclusions of this article are included within the article.
